# Introduction of the human *AVPR1A* gene substantially alters brain receptor expression patterns and enhances aspects of social behavior in transgenic mice

**DOI:** 10.1242/dmm.017053

**Published:** 2014-06-12

**Authors:** Rhonda Charles, Takeshi Sakurai, Nagahide Takahashi, Gregory A. Elder, Miguel A. Gama Sosa, Larry J. Young, Joseph D. Buxbaum

**Affiliations:** 1 Seaver Autism Center for Research and Treatment, Icahn School of Medicine at Mount Sinai, New York, NY 10029, USA; 2 Department of Genetics and Genomic Sciences, Icahn School of Medicine at Mount Sinai, New York, NY 10029, USA; 3 Department of Psychiatry, Icahn School of Medicine at Mount Sinai, New York, NY 10029, USA; 4 Department of Neurology, Icahn School of Medicine at Mount Sinai, New York, NY 10029, USA; 5 Neurology Service, James J. Peters VA Medical Center, Bronx, NY 10468, USA; 6 Research and Development Service, James J. Peters VA Medical Center, Bronx, NY 10468, USA; 7 Center for Translational Social Neuroscience, Department of Psychiatry and Behavioral Sciences, Yerkes National Primate Research Center, Emory University, Atlanta, GA 30329, USA; 8 Department of Neuroscience, Icahn School of Medicine at Mount Sinai, New York, NY 10029, USA; 9 Friedman Brain Institute and Mindich Child Health and Development Institute, Icahn School of Medicine at Mount Sinai, New York, NY 10029, USA

**Keywords:** AVPR1A, Humanized mouse, Social behavior, Species-specific, Microsatellite, Autism

## Abstract

Central arginine vasopressin receptor 1A (AVPR1A) modulates a wide range of behaviors, including stress management and territorial aggression, as well as social bonding and recognition. Inter- and intra-species variations in the expression pattern of AVPR1A in the brain and downstream differential behavioral phenotypes have been attributed to differences in the non-coding regions of the *AVPR1A* gene, including polymorphic elements within upstream regulatory areas. Gene association studies have suggested a link between *AVPR1A* polymorphisms and autism, and AVPR1A has emerged as a potential pharmacological target for treatment of social cognitive impairments and mood and anxiety disorders. To further investigate the genetic mechanism giving rise to species differences in AVPR1A expression patterns and associated social behaviors, and to create a preclinical mouse model useful for screening drugs targeting AVPR1A, we engineered and extensively characterized bacterial artificial chromosome (BAC) transgenic mice harboring the entire human *AVPR1A* locus with the surrounding regulatory elements. Compared with wild-type animals, the humanized mice displayed a more widely distributed ligand-AVPR1A binding pattern, which overlapped with that of primates. Furthermore, humanized *AVPR1A* mice displayed increased reciprocal social interactions compared with wild-type animals, but no differences in social approach and preference for social novelty were observed. Aspects of learning and memory, specifically novel object recognition and spatial relocation recognition, were unaffected. The biological alterations in humanized *AVPR1A* mice resulted in the rescue of the prepulse inhibition impairments that were observed in knockout mice, indicating conserved functionality. Although further behavioral paradigms and additional cohorts need to be examined in humanized *AVPR1A* mice, the results demonstrate that species-specific variations in the genomic content of regulatory regions surrounding the *AVPR1A* locus are responsible for differential receptor protein expression patterns across species and that they are likely to contribute to species-specific behavioral variation. The humanized *AVPR1A* mouse is a potential preclinical model for further understanding the regulation of receptor gene expression and the impact of variation in receptor expression on behaviors, and should be useful for screening drugs targeting human AVPR1A, taking advantage of the expression of human AVPR1A in human-relevant brain regions.

## INTRODUCTION

Arginine vasopressin (AVP) is a natural, endogenous ligand that binds to and activates AVPR1A in both the central and peripheral system. Vasopressin signaling in the brain through arginine vasopressin receptor 1A (AVPR1A) has been shown to be a key modulator of numerous behaviors, including pair-bonding, parental care, aggression and stress-coping (Egashira et al., 2009; Insel, 2010; Koshimizu et al., 2012; Meyer-Lindenberg et al., 2011). Pharmacological approaches and the evaluation of numerous animal models have proven useful for elucidating the role of AVPR1A in behavior (Appenrodt et al., 1998; Bielsky et al., 2004; Bielsky et al., 2005b; Ebner et al., 1999; Egashira et al., 2004; Egashira et al., 2007; Ferris et al., 2006; Liebsch et al., 1996; Lim et al., 2004; Wersinger et al., 2007). For example, administering selective Avpr1a antagonists resulted in reduced offensive aggressive behaviors in Syrian golden hamsters (Ferris et al., 2006) and had anxiolytic and anti-depressant effects in rats (Ebner et al., 1999; Liebsch et al., 1996). Furthermore, *Avpr1a* knockout mice displayed reduced anxiety, impaired reciprocal social interaction and decreased social recognition (Bielsky et al., 2004; Egashira et al., 2007), indicating that the receptor is an important factor in mediating essential social behaviors. Although AVPR1A has been associated with several pro-social and affiliative functions, in certain species and within specific contexts, it also mediates processes that might yield anti-social behaviors, including increased aggression and anxiety. Thus, blocking AVPR1A function in rats can actually increase social interactions, most probably by reducing social anxiety. All in all, given the structural and functional complexity of the ‘social behavior neural network’ and the potential contribution of other molecular and environmental factors in co-modulating a variety of social behaviors (Albers, 2012), the diverse effects associated with positive or negative regulation of AVPR1A are not surprising.

The expression pattern of AVPR1A in the brain differs considerably between and within species and is thought to be a major determinant that accounts for differences in certain behavioral traits (Donaldson and Young, 2008; Young et al., 1999a; Young et al., 1997). Specifically, extensive studies in voles have shown that species differences, as well as individual variation in AVPR1A expression in the brain, are linked to the diversity in social behavior between subspecies (Donaldson and Young, 2008; Lim et al., 2004; Pitkow et al., 2001; Young and Wang, 2004). For example, in comparison with non-monogamous vole species, monogamous prairie voles have higher densities of *Avpr1a* expression in the ventral pallidum (Insel et al., 1994; Young et al., 1997), and increasing Avpr1a expression in this region through viral vector gene transfer facilitates social bonding and partner preference formation in both male prairie voles and the non-monogamous meadow voles (Lim et al., 2004; Pitkow et al., 2001). Furthermore, transgenic mice harboring the prairie *Avpr1a* locus display ligand binding to AVPR1A in a pattern similar to that seen in prairie voles and also display increased pro-social behavior in response to an AVP challenge, as compared with wild-type mice (Young et al., 1999a).

**RESOURCE IMPACT**

**Background**

The *AVPR1A* gene encodes arginine vasopressin receptor 1A (AVPR1A), a G-protein coupled receptor expressed in the brain, liver, kidney and vasculature. There is overwhelming evidence supporting the role of AVPR1A-mediated vasopressin signaling in behavioral modulation. Specifically, studies have revealed associations between polymorphic microsatellites surrounding the gene and aggressive behaviors in borderline personality disorders and social deficits associated with autism spectrum disorders. Furthermore, vasopressin is elevated in major depression and is a regulator of hypothalamic-pituitary-adrenal axis function during chronic psychological stress. Although animal models have provided insight into the function of AVPR1A in behavior modulation, these models have more limited usefulness in evaluating therapeutics for the treatment of abnormal behaviors associated with psychiatric diseases because of variability in receptor expression patterns and pharmacology between species. A humanized mouse model is likely to be more useful in elucidating the mechanisms of gene regulation and gene expression that leads to specific behavioral phenotypes and could provide a suitable *in vivo* model for novel drug screens.

**Results**

The authors introduced the human *AVPR1A* gene and its regulatory elements into mice using BAC transgenesis. The resulting humanized mouse model was characterized using biochemical analyses and behavioral tests. The brain receptor distribution of the mice was reported to be significantly different from that of wild-type mice. Interestingly, this expression pattern overlaps with that of humans and primates, suggesting that differential genomic elements within the non-coding regions of the BAC insert influenced the expression of the receptor, in a species-specific manner. Furthermore, analysis of behaviors showed that the humanized mouse did not display the sensorimotor gating deficits observed in *Avpr1a* knockout mice. Compared with wild-type mice, the humanized mice demonstrated heightened social reciprocity with no changes to general memory.

**Implications and future directions**

The humanized AVPR1A model described here provides a new preclinical model for understanding the regulation of the receptor, its role in the central vasopressin system, its modulation of behaviors and the mechanism by which it leads to disease pathology. Although drugs targeting the vasopressinergic system and AVPR1A have been extensively studied, pharmaceutical companies have historically focused on developing compounds with relevance to the peripheral physiology. More recent preclinical and clinical studies in animal models and human subjects have demonstrated that this receptor subtype is a particularly relevant target for treatment of psychiatric illnesses, including autism and mood and anxiety disorders. This study provides evidence that further reinforces the rationale for the development of drugs that modulate the central vasopressin system and AVPR1A signaling and also provides a model that can be used to appropriately test candidate therapeutics.

Species differences in ligand binding to Avpr1a have been associated with variation in a polymorphic microsatellite in the non-coding 5′-flanking region of the vole *Avpr1a* locus (Hammock et al., 2005; Hammock and Young, 2004; Hammock and Young, 2005). Furthermore, differences in microsatellite structure within the prairie vole species have been associated with variation in pair-bonding behavior between individual animals (Hammock and Young, 2002; Hammock and Young, 2004). The proximal 5′-flanking region of the *Avpr1a* gene is not the only factor to direct species-typical expression profiles as transgenic mice in which homologous recombination was used to replace 3.5 kb of the murine *Avpr1a* proximal 5′-flanking region with the homologous prairie vole sequence expressed Avpr1a largely in a mouse pattern, indicating that more distal chromosomal elements are necessary to confer species-specific expression patterns (Donaldson and Young, 2013).

As in rodents, analogous polymorphic regions upstream of the human *AVPR1A* coding sequence have been the focus of investigation into the diverse behavioral phenotypes displayed in both normal and psychiatric populations (Bachner-Melman, 2005; Bachner-Melman et al., 2005; Caldwell et al., 2008; Donaldson and Young, 2008; Hammock and Young, 2005; Israel et al., 2008; Meyer-Lindenberg et al., 2009). Studies have revealed an association between polymorphic microsatellites RS1 and RS3 and measures of perceived power, closeness and conflict between siblings (Bachner-Melman, 2005). Polymorphisms in *AVPR1A* have also been associated with altruism (Israel et al., 2008; Knafo et al., 2008) and pair-bonding behavior in men, as measured by perceived partner bonding, marital status, as well as spousal perception of marital quality (Walum et al., 2008). Furthermore, in normal populations, differences in the length of RS3 are shown to be associated with altered gene function. Specifically, analyses of post-mortem hippocampal samples demonstrate that long forms of the RS3 polymorphism are associated with increased mRNA expression (Knafo et al., 2008) and a functional magnetic resonance imaging study shows that RS3 polymorphism length is a determinant of amygdala activation in an emotional face-matching task (Meyer-Lindenberg et al., 2009).

In individuals with borderline personality disorder, the RS3 microsatellite has been reported to be associated with measures of impulsive aggression (Vogel et al., 2012). Additionally, variations in the RS3 polymorphism have been associated with differing levels of prepulse inhibition (Kohl et al., 2013; Levin et al., 2009), an autonomic response that is often deregulated in disorders of social cognition, including schizophrenia. Multiple association studies support a link between RS1, RS3 and single nucleotide polymorphisms (SNPs) in the upstream flanking region of *AVPR1A* and autism spectrum disorders (Kim et al., 2002; Tansey et al., 2011; Wassink et al., 2004; Yang et al., 2010; Yirmiya et al., 2006), and the association might be partly mediated by deficits in social skills (Yirmiya et al., 2006). Collectively, these studies show that genetic variability within the *AVPR1A* locus can be an important determinant of social cognitive behavior. In addition, vasopressin has been shown to be elevated in individuals with major depression and has also been identified as a primary regulator of hypothalamic-pituitary-adrenal axis (HPA) function during chronic psychological stress (Simon et al., 2008; van Londen et al., 1997; Volpi et al., 2004). Given the overwhelming evidence supporting the role of AVPR1A-mediated vasopressin signaling in behavior modulation, ongoing studies have focused on developing pharmacotherapeutic interventions directed at the receptor for the treatment of debilitating global health problems, such as anxiety, depression, autism and other behavioral disorders (Ring, 2005; Ryckmans, 2010; Simon et al., 2008).

In addition to species differences in AVPR1A distribution and ligand binding profiles, rodent and primates display differential selectivity for and responsiveness to AVP and synthetic agonists or antagonists (Manning et al., 2012). Such factors, distinguishing mouse and human AVPR1A structure and function, present a challenge to the *in vivo* evaluation of therapeutic compounds targeted to humans, in rodent models. In order to examine the role of proximal and distal surrounding sequences on variation in gene expression patterns and to create a preclinical mouse model for screening human-specific AVPR1A drugs and their molecular and behavioral effects, we created a bacterial artificial chromosome (BAC) transgenic mouse harboring a >180-kbp fragment that included *AVPR1A* and the surrounding genomic context.

We hypothesized that the differential regulation of species-specific genomic elements within the non-coding, non-conserved region of the BAC transgene would result in an altered and potentially primate-like protein expression pattern in humanized animals in comparison with wild-type animals. Based on the findings of previous studies, which elucidated the behavioral roles of AVPR1A, we further hypothesized that alterations of AVPR1A in humanized animals in comparison with wild-type and knockout mice would be accompanied by predictable differences in social behavior, memory and sensorimotor gating. Although we have not yet parsed the degree to which behavioral changes in humanized animals are due to altered receptor binding affinities, expression levels and/or distribution patterns, this model might serve as a useful investigatory preclinical tool, while providing opportunities for further dissection of the social behavior neural network.

## RESULTS

### *AVPR1A* BAC transgenic animals

**Fig. 1.**
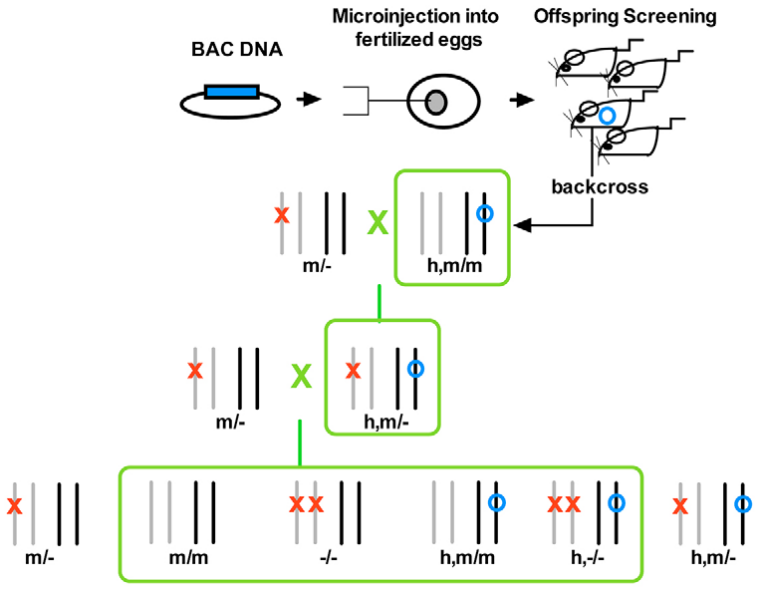
**Generation of BAC transgenic animals.** A BAC transgenesis approach was employed to generate founder mice that incorporated a human *AVPR1A*-containing BAC into the mouse genome. The breeding scheme ultimately generated the four genotype groups of interest (m/m, wild type; −/−, knockout; h,m/m, partially humanized; h,−/−, fully humanized; indicated by the lower green box) to be used in biochemical and behavioral analyses. Gray and black pairs of lines represent two different pairs of chromosomes. The red X represents a knockout of the murine *Avpr1a* gene and the blue circle represents an insertion of the human transgene (without any specification of a specific chromosome).

We generated two lines of the humanized AVPR1A BAC transgenic animals (Fig. 1). Both lines were used in ^125^I radioligand assays and, observing no differences in AVPR1A expression between the two lines (see below), line 1 animals were then used for all subsequent biochemical analyses and behavioral tasks.

To characterize the expression of the human *AVPR1A* gene in our transgenic mice, we first confirmed that *AVPR1A* mRNA was expressed in brain of line 1 mice. Comparison of quantitative real-time reverse-transcriptase (qRT)-PCR results for mRNA extracts from the left hemisphere of brains of 6-month-old wild-type (m/m) and partially humanized (h,m/m) mice showed that the introduction of the human transgene did not affect the levels of mouse-specific *Avpr1a* mRNA (*t*-test, *P*=0.212; supplementary material Fig. S1A). However, there was strong expression of human *AVPR1A* mRNA in humanized animals. In a comparison between partially (h,m/m) and fully humanized (h,−/−) animals, we observed that the expression level of human AVPR1A was unaffected by the presence or absence of the endogenous murine *Avpr1a* (*t*-test, *P*=0.436; supplementary material Fig. S1B).

Given the overlap between the vasopressin and oxytocin systems, we also measured the expression of endogenous oxytocin receptor (*Oxtr*), vasopressin (*Avp*) and oxtocin (*Oxt*) and showed that there was no significant difference in the expression of these genes across genotypes (supplementary material Fig. S1C) [*Oxtr: F*_(3,24)_=0.269, *P*=0.847; *Avp: F*_(3,24)_=0.718, *P*=0.551; *O*xt: *F*_(3,24)_=0.402, *P*=0.753; one-way ANOVA].

### AVPR1A protein distribution

**Fig. 2.**
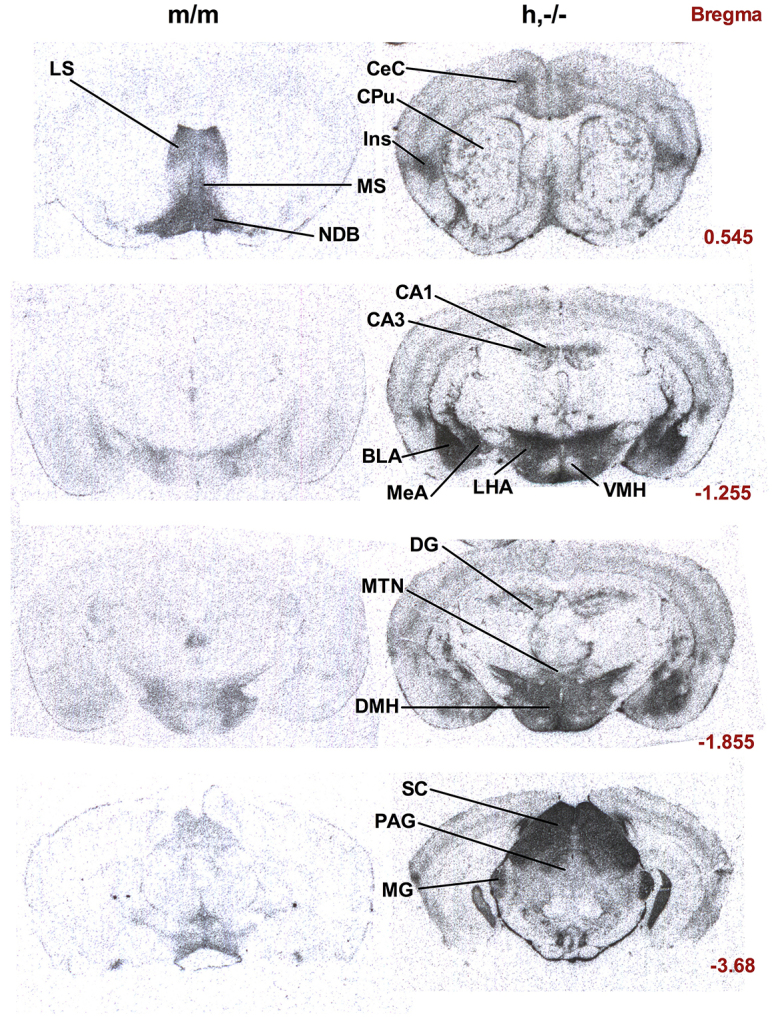
**Human and mouse AVPR1A protein distribution pattern in the brains of transgenic animals.** Radioactive AVP-I^125^ ligand binding was performed on fresh coronally-cut slide-mounted sections of m/m (*n*=10), −/− (*n*=6) (not shown), h,m/m (*n*=10) (not shown) and h,−/− (*n*=6) mice. Representative sections from the developed film were compared between genotypes. LS, lateral septum; MS, medial septal nucleus; NDB, nucleus of diagonal band; CeC, cerebral cortex; CPu, caudate putamen; Ins, insular cortex; CA1, field CA1, Ammon’s horn of hippocampus; CA3, field CA3, Ammon’s horn of hippocampus; BLA, basolateral nucleus of the amygdala; MeA, medial nucleus of the amygdala; LHA, lateral hypothalamic area; VMH, ventromedial nucleus of the hypothalamus; DG, dentate gyrus; MTN, midline thalamic nuclei; DMH, dorsomedial hypothalamic nucleus; SC, superior colliculus; PAG, periaqueductal gray; MG, medial genticulate nucleus. Numbers to the right of the figure indicate the distance of the section from a coronal plane passing through the bregma.

**Fig. 3.**
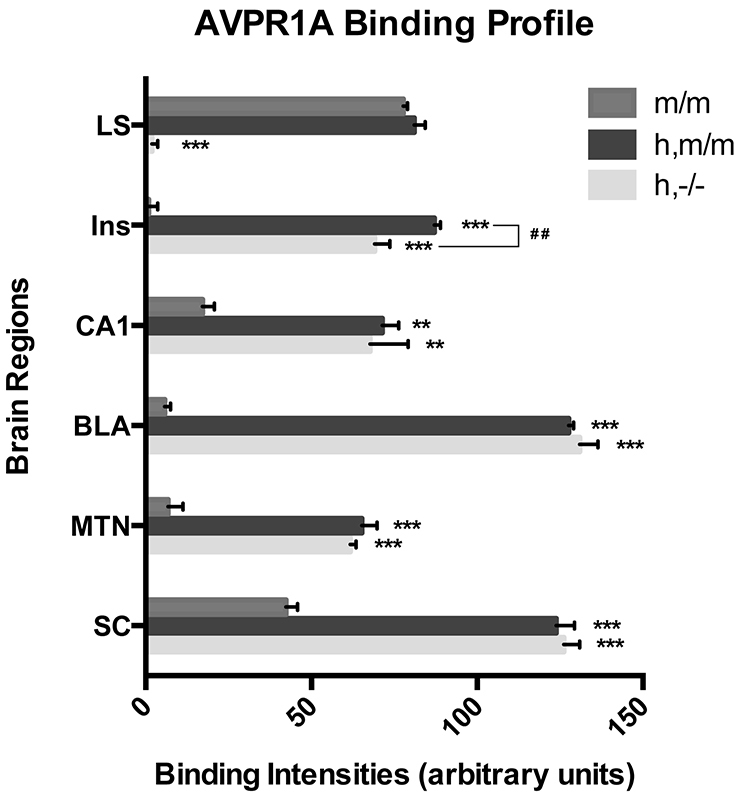
**Human and mouse AVPR1A protein levels quantified for specific brain regions.** Human and mouse AVPR1A binding intensities for specific regions were quantified using ImageJ software from the indicated genotypes. LS, lateral septum; Ins, insular cortex; CA1, field CA1, Ammon’s horn of hippocampus; BLA, basolateral nucleus of the amygdala; MTN, midline thalamic nuclei; SC, superior colliculus. **P<0.01, ***P<0.001 using one-way ANOVA analysis with comparison to m/m animals. ^##^*P*<0.01 from comparisons between h,m/m and h,−/− animals. Data represent the group mean±s.e.m.

To identify differences in protein expression of human and mouse AVPR1A between genotypes, we employed AVP-I^125^ radioligand binding, a reliable assay that is commonly used to map the distribution of functional human and mouse AVPR1A receptors with exceptional anatomical resolution (alternative techniques for identifying protein expression levels and distribution, such as western blotting and immunohistochemistry, proved unsuitable due to the lack of validated, commercially available antibodies against AVPR1A). In line 1 animals, transgenic mice expressing the human form of *AVPR1A* showed profoundly different binding patterns when compared with wild-type littermates (Figs 2, 3), a finding that was replicated in the second, independently-derived line (supplementary material Fig. S2).

Binding levels were quantified for specific brain regions in wild-type (m/m), knockout (−/−), overexpressing (h,m/m) and fully humanized (h,−/− animals). Knockout animals did not demonstrate significant receptor-ligand binding above background levels and were excluded from subsequent statistical tests. A one-way ANOVA analysis showed that each of the analyzed brain regions demonstrated significant overall genotype effects (lateral septum, basolateral nucleus of the amygdala, insular cortex, superior colliculus, midline thalamic nuclei each *P*<0.001; field CA1 Ammon’s horn of hippocampus *P*=0.004) and post-hoc least significant difference tests highlighted the significance of individual genotype comparisons (Fig. 3). In wild-type animals (m/m), ligand binding was observed in previously documented areas, including the lateral septum, bed nucleus of stria terminalis, superior colliculus, substantia nigra and dorsal raphe (Dubois-Dauphin et al., 1996; Johnson et al., 1993; Tribollet et al., 1991) (see supplementary material Table S1). In fully humanized animals (h,−/−), ligand binding was more intense than wild-type animals and widely distributed to areas in which primate AVPR1A is highly expressed, including the midline thalamic nucleus, striatum, and regions of the brain stem and spinal cord (Loup et al., 1991; Young et al., 1999b). As in the rhesus macaque, there was significant binding of AVP in amygdala nuclei and insular cortex of the humanized animals. Additionally, strong binding was observed in the hippocampus (including the CA1, CA3 and dentate gyrus), a region in which *AVPR1A* mRNA has previously been shown to be highly transcribed in humans (Knafo et al., 2008). In contrast to human and macaque, the h,−/− animals did not demonstrate significant binding beyond background levels in the lateral septum (Fig. 3).

The h,m/m mouse receptor expression distribution demonstrates an apparent overlay of the m/m and h,−/− pattern. For example, lateral septum expression of AVPR1A was absent in the h,−/− animal, whereas the h,m/m animal showed levels of binding that were comparable to those of the m/m animal in this region. The same pattern of expression was observed in the nucleus of the diagonal band (Fig. 2; supplementary material Fig. S2). This supports our finding (supplementary material Fig. S1) that introduction of the human transgene does not affect the expression of murine *Avpr1a* and confirms that the human *AVPR1A* transgene is both expressed more widely, as well as being differentially expressed in specific brain regions. Comparison of h,m/m and h,−/− animals demonstrated that AVPR1A binding, in all but one of the quantified regions, was unaffected by the presence or absence of the murine AVPR1A protein (Fig. 3).

Additionally, results of an OXTR-ligand binding assay that was performed on adjacent sections showed no significant differences in OXTR binding between our genotypes (data not shown).

### Reciprocal social interaction

**Fig. 4.**
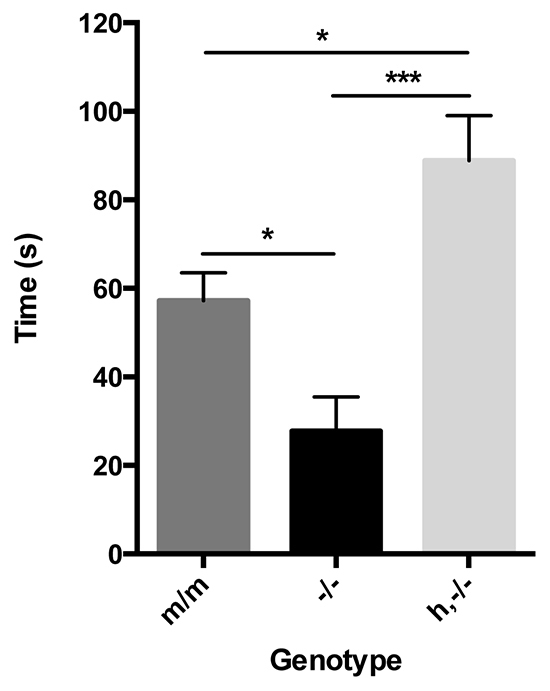
**Adult reciprocal social interaction.** Mice (m/m, *n*=9; −/−, *n*=11; h,−/−, *n*=15) were scored for time spent in social interaction (sniffing, grooming and close following) with a conspecific male partner over a 10-minute period. *P<0.05, ***P<0.001 using one-way ANOVA analysis. Data represent the group mean±s.e.m.

Both mouse and human AVPR1A have been shown to have an important role in regulating normal social behaviors. In addition, our ligand-binding assays identified receptor expression differences in regions that are important for modulating aspects of social interaction. To evaluate the effects of AVPR1A expression in reciprocal social interactions, we used a trial in which male test animals were placed with a conspecific unfamiliar male partner and sniffing, grooming and close following behaviors displayed by the test animal were quantified. This trial was chosen based on previous findings of a deficit in *Avpr1a* knockout mice (Egashira et al., 2007). We confirmed that knockout animals showed significantly lower levels of social interaction with partner animals when compared with wild-type animals (*P*=0.026, one-way ANOVA; Fig. 4). Interestingly, h,−/− animals showed significantly heightened interaction with partner animals when compared with both knockout and wild-type animals (*P*<0.001 and *P*=0.012, respectively, overall genotype effect *F*_(2,32)_=15.239, *P*<0.001; one-way ANOVA), indicating that, in this test involving fully reciprocal social interactions, the insertion of human AVPR1A increases overall sociability. Analysis of the time spent engaged in non-social activity, such as rearing and self-grooming, revealed no significant differences between genotypes (data not shown).

To investigate other aspects of social behaviors, we made use of the three-chamber task. This choice-based, two-part trial measured the test animal’s preference for a novel object versus a novel animal during the ‘social approach’ portion (supplementary material Fig. S3A,B), and in the subsequent ‘preference for social novelty’ portion, test animals were given a choice of interacting with a novel animal versus a familiar animal (supplementary material Fig. S3C,D). Knockout animals showed deficits in social behaviors for both portions of the three-chamber test. Remarkably, the insertion of the human transgene in the h,−/− animals did not rescue the impairments in social approach or preference for social novelty.

### Object recognition tasks

**Fig. 5.**
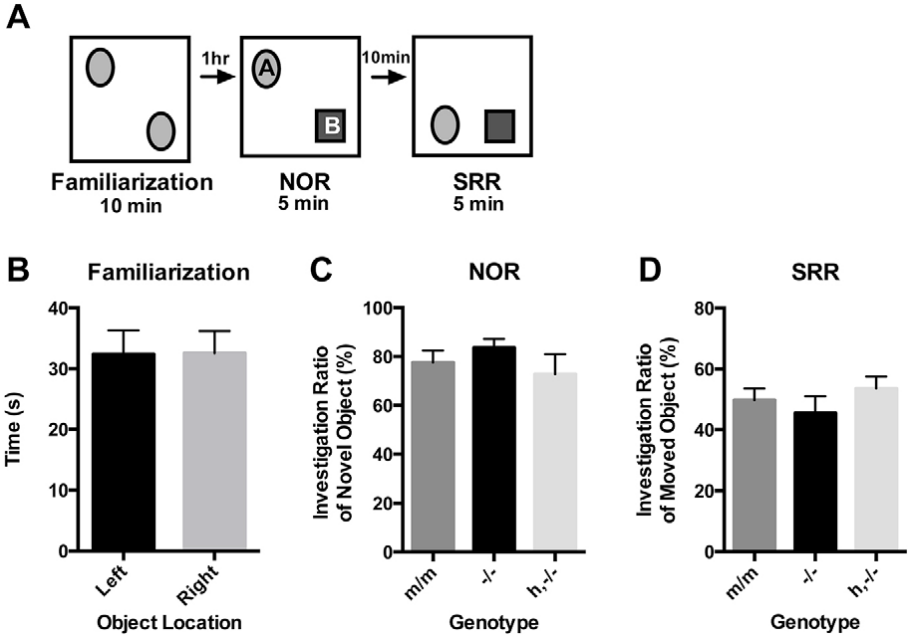
**Object recognition tasks.** (A) Mice (m/m, *n*=8; −/−, *n*=10; h,−/−, *n*=10) were subjected to a test comprising three trials, which were designed to evaluate the mouse’s memory of objects and of spatial localization. (B) During the familiarization trial, animals were scored for time spent investigating two identical objects. (C) Animals were scored for the amount of time they spent investigating the novel object during the NOR task and (D) for the amount of time spent investigating the object that had been moved during the SRR component, and investigation ratios are represented. The investigation ratio for the NOR test was determined by B_time_/(A_time_+B_time_) and the SRR investigation ratio was determined by A_time_/(A_time_+B_time_) for each subject. Statistical analyses made use of ANOVA and data represent mean±s.e.m.

Given the evidence for altered receptor expression in memory-associated brain regions, we performed novel object recognition and spatial relocation recognition tasks (Fig. 5A) to determine whether there were alterations to general memory and learning. No significant differences in these measures were detected between genotypes (Fig. 5B–D) [novel object recognition (NOR): *F*_(2,25)_= 0.897, *P*=0.42; spatial relocation recognition (SRR): *F*_(2,25)_=0.779, *P*=0.47].

### Sensorimotor gating

**Fig. 6.**
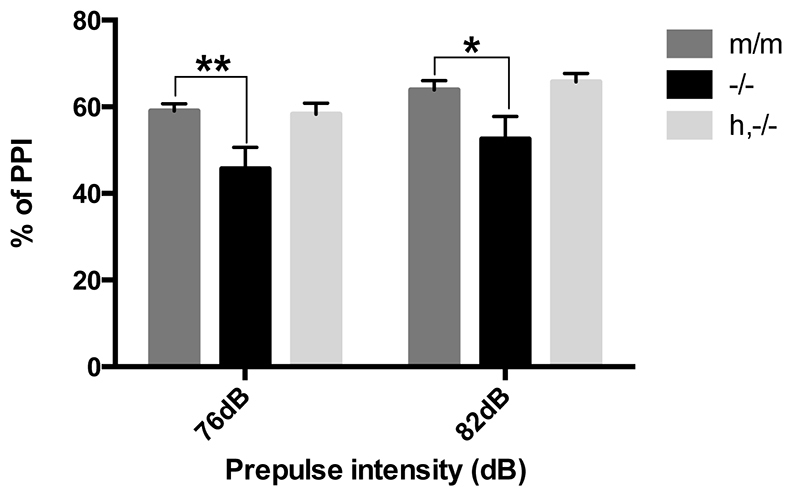
**Prepulse inhibition of startle.** The percentage of PPI in three genotypes (m/m, *n*=18; −/−, *n*=14; h,−/−, *n*=14) at prepulse intensities of 76 and 82 dB are shown. *P<0.05, **P<0.001 from two-way ANOVA with repeated measures analysis followed by one-way ANOVA analysis of individual prepulse intensities. Data represent mean±s.e.m.

Deficits in prepulse inhibition (PPI), a phenomenon in which a weak pre-stimulus attenuates an animal’s startle response to a subsequent larger stimulus, are correlated with impairments of sensorimotor gating. Although previous studies of *Avpr1a* knockout animals are conflicting, with one study demonstrating no changes in PPI and another showing deficits in PPI, we speculated that the loss of AVPR1A expression in important regions of the sensorimotor gating circuitry justified the re-examination of this phenomenon in knockouts. Furthermore, because humanized mice showed altered ligand binding in multiple regions within this circuitry, we hypothesized that any impairment in sensorimotor gating could be rescued by the introduction of the human *AVPR1A* transgene. Analyses of PPI in our mice by using a two-way ANOVA with repeated measures showed a genotype effect (*F*_(2,41)_=4.314, *P*=0.019), a decibel (dB) effect (*F*_(3,123)_=24.5, *P*<0.001), as well as a genotype with decibel interaction (*F*_(6,123)_=2.799, *P*=0.014). Specifically, subsequent one-way ANOVA tests at individual prepulse levels showed that for prepulse intensities of 76 dB and 82 dB, knockout animals demonstrated a significant deficit in PPI percentage when compared with that of wild-type animals (m/m vs −/−, 76 dB: *P*=0.003; 82 dB: *P*=0.014) (Fig. 6). However, in humanized animals, this impairment was effectively rescued to levels that were statistically comparable to that of the wild-type animal (m/m vs h,−/−, 76 dB: *P*=0.864; 82 dB: *P*=0.682).

## DISCUSSION

### Humanized AVPR1A BAC transgenic animals

Multiple studies have suggested that differences in region-specific expression patterns and levels of mouse or human AVPR1A are mediated by polymorphic elements in the proximal 5′-flanking region (Bachner-Melman, 2005; Hammock et al., 2005; Hammock and Young, 2004). However, a recent study in which a 3.5-kb region of the proximal 5′-flanking region of the mouse *Avpr1a* was replaced with prairie vole sequence suggests that species-typical expression patterns are also determined, to a large degree, by other factors (Donaldson and Young, 2013). In support of this, our BAC transgenic mice harboring 109 kb of upstream sequence and 64 kb downstream sequence displayed strong differences in AVPR1A receptor expression, consistent with the hypothesis that multiple proximal and distal chromosomal regulatory elements contribute to robust species differences in *AVPR1A* and *Avpr1a* expression patterns. Although we cannot be sure that the expression patterns seen in our two founder lines are identical to human expression patterns, there are some clear parallels with expression patterns that have been seen previously in rhesus macaque and human.

Our qRT-PCR analysis confirmed that the human *AVPR1A* transcript was abundantly expressed in the humanized mice, indicating that the altered receptor binding patterns were due to expression of the human allele. The inclusion of the transgene did not affect the expression levels of endogenous *Avpr1a* (supplementary material Fig. S1A) or other genes in the vasopressin system, such as *Oxtr*, *Oxt* and *Avp* (supplementary material Fig. S1C); similarly, the levels of human *AVPR1A* mRNA were unaffected by the presence or absence of the murine gene (supplementary material Fig. S1B), indicating that the transgene and murine genes are independently regulated and do not create compensatory expression effects in other genes involved in the vasopressin signaling system. This assured us that any changes in the behaviors observed in fully humanized mice were attributed to human *AVPR1A* gene expression and its differential expression levels and distribution in the brain.

Radioligand labeling was performed to map the protein expression patterns of functional human and mouse AVPR1A receptors, and the resulting ligand-binding patterns in transgenic mice demonstrated that the human AVPR1A protein was expressed at high levels in a pattern profoundly divergent from that of the murine AVPR1A protein. In control animals, as expected, ligand binding was observed in previously documented areas, with expression concentrated in the lateral septum, ventral pallidum, bed nucleus of the stria terminalis and anterior hypothalamus (Dubois-Dauphin et al., 1996; Johnson et al., 1993; Tribollet et al., 1991). In humanized BAC transgenic animals, high levels of binding in most of the aforementioned areas was accompanied by strong binding to AVPR1A throughout the cerebral cortex and regions of the brain stem, in a pattern overlapping with that of rhesus macaques. Furthermore, in comparison with control animals, the levels of binding were profoundly increased in the thalamus, amygdala and hippocampus of transgenic mice and in this way, paralleled the regional *AVPR1A* expression pattern observed in the human brain (Figs 2, 3; supplementary material Table S1). Curiously, although rhesus macaque and human both display AVPR1A binding in the septum, our mice did not express the human receptor in this region, which might be attributed to differential expression of *trans* regulatory elements between primate and mouse.

As in the analysis of qRT-PCR results, our comparisons of mouse and human AVPR1A-ligand binding in the h,m/m, m/m and h,−/− mice demonstrated that the inclusion of the human transgene did not appear to affect the expression of the murine AVPR1A protein. Additionally, these comparisons demonstrated that the expression levels and distribution of the human protein was generally unaffected by the presence or absence of the endogenous protein. This further supports the idea that the human transgene and endogenous *Avpr1a* are independently regulated in humanized animals. Furthermore, the consistency of the OXTR-ligand binding results across genotypes indicated that genetically induced alterations in the expression of murine and/or human AVPR1A did not affect the expression of other components of the vasopressin system or induce compensatory effects.

### Social behaviors

A previous study utilizing a trial that allowed free-moving reciprocal social investigation between test and partner animals demonstrated that AVPR1A was important in establishing normal social interactions (Egashira et al., 2007). Furthermore, transgenic mice expressing the prairie vole *Avpr1a* gene, which demonstrated a more widely distributed receptor pattern similar to that seen in prairie voles, displayed increased affiliative behaviors in response to AVP administration (Young et al., 1999a). Therefore, we compared social interactions between *Avpr1a* knockout, m/m and h,−/− mice in an arena in which the mice could move freely (Fig. 4). We confirmed the expected reduction in social interactions in the knockout animals compared with wild-type littermates, and, most excitingly, the humanized mice displayed a significant increase in social interactions. These findings indicated that the expression of human AVPR1A in our lines was in areas that promote certain aspects of social interaction and that this maps to regions that normally do not express AVPR1A in wild-type mice. The important role of AVPR1A in modulating reciprocal social behavior is further supported by the observation that general non-social activity was unaffected between groups, for this particular test.

We also performed the three-chamber social approach trial in which test subjects were given a choice between a novel object and novel animal and for which the results are thought to be indicative of a specific domain of sociability. The social approach task revealed a deficit in social approach in knockout animals and the introduction of the human *AVPR1A* transgene did not rescue this impairment (supplementary material Fig. S3A,B). This finding is in contrast to the findings of the reciprocal social interaction task and indicates that the altered AVPR1A expression in the brain of transgenic animals does not affect behaviors that are relevant in some measures of social approach. The contrasting results might be related to differences in experimental design because, in the three-chambered social approach test, elements of social reciprocity are missing. Human AVPR1A also did not rescue preference for social novelty (supplementary material Fig. S3C,D). Previous studies in mice revealed that *Avpr1a* expression in the lateral septum is necessary and sufficient for social recognition (Bielsky et al., 2005a; Egashira et al., 2004). Thus, the lack of preference for social novelty could conceivably be due to the lack of expression of human AVPR1A in the lateral septum of h,−/− animals.

Our results indicate that although the humanized mouse rescued the deficits in reciprocal social behaviors observed in knockouts, other deficits corresponding to additional components of social behavior were unaltered. Further validation of this mouse model is required in order to more accurately define the role of human AVPR1A in modulating overall social behavior. Future studies should employ alternative behavioral tests that address the structural and functional complexity of the ‘social behavior neural network’ by analyzing other aspects of social behavior.

### Memory and learning

Given the significant increase in ligand binding in the hippocampus of the humanized animal (Figs 2, 3), we used object recognition tasks to test memory in our mice (Fig. 5). Previous studies have shown that *Avpr1a* knockout animals show deficits in the eight-arm radial maze but not in the Morris water maze (Egashira et al., 2004). Because the Morris water maze elicits anxiety and stress responses, and the eight-arm radial maze utilizes an external motivational reward system, we decided to perform simple object recognition tasks to measure memory, preference for novelty and spatial learning. Our results demonstrated that neither the novel object nor spatial relocation recognition tasks yielded significant differences between all genotypes, including the knockout group (Fig. 5C,D). The results of this particular test suggest that changes in social behaviors in knockout and transgenic animals are not due to non-social recognition memory deficits, and, conversely, expression changes in the brain of transgenic animals might not have any clear effect on the specific aspects of learning and memory that are tested in this trial. Again, follow-up studies utilizing alternative experimental designs and additional cohorts are required to definitively outline the role of mouse and human AVPR1A in multiple aspects of memory and learning.

### Sensorimotor processing

Because one rationale for creating humanized *AVPR1A* mice was to develop an animal model useful for screening drugs targeting AVPR1A, we needed to determine whether human AVPR1A was functionally coupled to second messenger effector systems. Because human and rodent AVPR1A display different expression and pharmacological binding profiles, a humanized mouse model would be superior to a wild-type mouse for many such studies. Sensorimotor processing is modulated by an extensive circuitry, which includes parts of the thalamus, basal ganglia and cortex, regions which demonstrated increased AVPR1A-ligand binding in humanized animals. Deficits in PPI, a hallmark measure of sensorimotor gating, have been frequently reported in schizophrenia (Braff et al., 2001) and, more recently, in autism (Kohl et al., 2013). Moreover, studies have shown that variations in the polymorphic region of the *AVPR1A* gene are linked to PPI levels (Levin et al., 2009). Previous animal model studies investigating the role of *Avpr1a* in sensorimotor processing have been contradictory (Bielsky et al., 2004; Egashira et al., 2006). In our studies, we identified a significant deficit in PPI in knockout animals as compared with wild-type controls, an impairment that was restored to normal levels in humanized animals (Fig. 6). Although these results need to be replicated in additional cohorts, these first findings indicate that human AVPR1A was functionally coupled to the appropriate effector systems and second messenger cascade in the mouse brain.

### Conclusions

We documented strong and consistent regional alterations in AVPR1A protein expression in humanized animals. Although not perfectly consistent with human AVPR1A expression patterns, there are significant common features with expression in rhesus macaque and human, including widespread cortical, hippocampal and thalamic expression. In agreement with previous studies, our results indicated that more distal regulatory elements contribute to diversity in *AVPR1A* and *Avpr1a* expression levels and distribution patterns. The observation that human *AVPR1A* rescued sensorimotor gating deficits in *Avpr1a* knockout animals provides the first evidence that our humanized mice might be useful for screening drugs targeting AVPR1A for psychiatric disorders and potential therapies for depression and violence.

Interestingly, the deficits in free-moving reciprocal social behavior observed in knockout animals were not only rescued by the insertion of the human transgene, but humanized *AVPR1A* mice displayed enhanced reciprocal social interactions in this test. Although further behavioral investigation using alternative experimental tests is required, these results indicate that humanized mice might be useful for exploring how variation in *AVPR1A* and *Avpr1a* expression generates diversity in social behaviors.

## MATERIALS AND METHODS

### Generation of BAC transgenic mice and breeding scheme

A 182,910-bp BAC clone containing the entire coding sequence for the human *AVPR1A* gene was identified (RP11-715H19, GenBank no. AC025525) and used to create humanized *AVPR1A* mice. The BAC clone contained 109 kb upstream of the transcription start site and 64 kb downstream of the 3′ untranslated region. BAC DNA was partially purified by using Hirt precipitation followed by isopropanol precipitation, resuspension and digestion with an ATP-dependent exonuclease to remove any remaining contaminating bacterial genomic DNA. The resulting BAC DNA was further purified by ionic exchange chromatography, followed by a gentle phenol-chloroform extraction and ethanol precipitation. The BAC DNA was resuspended in 10 mM Tris-HCl, pH 7.6, 1 mM EDTA and then diluted to a concentration of 0.3 ng/μl in 30 nM spermine, 70 nM spermidine, 0.1 M NaCl. This preparation was used for microinjection into the pronucleus of fertilized B6C3 mice (C57BL/6xC3H mouse hybrid). Eggs surviving injection were transplanted into pseudopregnant females, and four of 22 birthed pups were confirmed to be transgene-positive by using PCR amplification of a human-specific genomic segment.

Two independent lines, lines 1 and 2, were generated by selective breeding in the format described in Fig. 1. BAC founders were backcrossed to wild-type C57BL6 mice for at least eight generations and to a minimum of 90% C57BL6 genomic background, as determined by Max-Bax Technology SNP marker genotyping (Charles River Laboratories, Wilmington, MA, USA). BAC-positive progeny were then crossed to heterozygous *Avpr1a* knockout animals (m/−) to obtain BAC-positive animals that were heterozygous for the knockout alleles. Hemizygous transgenic-heterozygous knockout mice (h,m/−) were then crossed to heterozygous knockout animals to obtain genotypes of interest: wild-type (m/m), knockout (−/−) and humanized mice (h,−/− and h,m/m) in the ratio of 1:1:1:1. To confirm that the transgenic mice incorporated the human gene into the murine genome, we continually performed PCR specifically targeting human *AVPR1A* using genomic DNA isolated from mouse tails and the following primers: forward, 5′-GGCGCTGGCAACACAAG-3′; and reverse, 5′-AGCACATTTGCGGCAGCACCT-3′. In order to differentiate between the knockout and wild-type animals, we performed genotyping using primers specific to the LacZ insert that was used to create the knockout animal (forward, 5′-GCGGTAGGTGATGTCCCAGCACAGC-3′; and reverse, 5′-GGGCCAGCTCATTCCTCCCACTCAT-3′), as well as primers that exclusively recognized murine *Avpr1a* (forward, 5′-GCGG-TAGGTGATGTCCCAGCACAGC-3′; and reverse, 5′-CGCAACGAGG-AGCTGGCGAAGCTGG-3′).

Hemizygous transgenic-heterozygous knockout mice (h,m/−) from line 1 have been made available through The Jackson Laboratory as stock number 025101.

### RNA isolation and qRT-PCR

Brains from 6-month-old male animals of line 1 (*n*=6 for each genotype) were dissected out, cut sagittally with the left-brain hemisphere being used for RNA extraction. Total RNA was prepared using the RNeasy miniprep protocol (Qiagen, Valencia, CA, USA), stored at −80°C, quantified using a spectrophotometer and assessed for quality and degradation using the Bioanalyzer Lab-on-a-chip (Agilent, Santa Clara, CA, USA). Total RNA was reverse transcribed into single-stranded cDNA using High Capacity cDNA Reverse Transcription kit (Applied Biosystems, Carlsbad, CA, USA). Quantitative PCR was performed using Universal Probe Library system (Roche, Indianapolis, IN, USA) and specially designed primers (Invitrogen, Carlsbad, CA, USA) to measure expression of both human and mouse *AVPR1A/Avpr1a*, as well as other genes of interest (listed below). Relative expression levels of genes of interest were normalized to control gene expression levels (*Gapdh* and *ActB*) using qBase software now available from Biogazelle (Ghent, Belgium). Primer sequences and Universal Probe Library probe numbers were mouse *Avpr1a*, no. 31, forward 5′-GGGATACCAATTTCGTTTGG-3′, reverse 5′-AAGCCAGTAACGCCG-TGAT-3′; human *AVPR1A*, no. 3, forward 5′-TTGTGATCGTGACGGC-TTAC-3′, reverse 5′-GATGGTAGGGTTTTCCGATTC-3′ and no. 9 forward 5′-ATCCCATGTCCGTCTGGA-3′, reverse 5′-TCAAGGAACCCAGTAAT-GCAG-3′; *Oxtr*, no. 29, forward 5′-GTGCAGATGTGGAGCGTCT-3′, reverse 5′-GTTGAGGCTGGCCAAGAG-3′; *Avp*, no. 40, forward 5′-CTACGCTCTCCGCTTGTTTC-3′, reverse 5′-GGGCAGTTCTGGAAGT-AGCA-3′; *Oxt*, no. 27, forward 5′-CACCTACAGCGGATCTCAGAC-3′, reverse 5′-CGAGGTCAGAGCCAGTAAGC-3′; *Gapdh*, no. 29, forward 5′-GCCAAAAGGGTCATCATCTC-3′, reverse 5′-CACACCCATCACAAAC-ATGG-3′; *ActB*, no. 63, forward 5′-ACTGCTCTGGCTCCTAGCAC-3′, reverse 5′-CCACCGATCCACACAGAGTA-3′.

### Receptor autoradiography

Radioligand receptor binding was performed to analyze both the distribution and density of both mouse and human AVPR1A protein in transgenic animals from lines 1 and 2. Six-month-old male animals (m/m, *n*=10; h,m/m, *n*=10; h,−/−, *n*=7; −/−, *n*=6) were rapidly decapitated, and the brains removed and frozen on dry ice. Forzen sections that were 20-μm thick were prepared by cutting on a cryostat, and seven serial sets of adjacent sections were made. For binding assays, slides were initially thawed and then fixed in 0.1% paraformaldehyde. After pre-incubation in 50 mM Tris, slides were exposed for 60 minutes to 50 pM ^125^I-lin-vasopressin (Perkin Elmer, Wellesley, MA, USA) in 50 mM Tris-MgCl_2_ and 0.1% bovine serum albumin. For OXTR binding assays, 50 pM ^125^I-labeled d(CH_2_)_5_[Tyr(Me)_2_,Tyr9-NH_2_] ornithine vasotocin (Perkin Elmer) in the same solution was used instead of the effective ligand. Following incubation, sections were washed extensively with 50 mM Tris-MgCl_2_ to reduce background. Nonspecific binding was defined in adjacent sections by co-incubation with 50 μM of unlabeled β-mercapto-β,β-cyclopentamethylenepropionyl 1, O-me-Tyr2, Arg8]-vasopressin (Perkin Elmer) or 50 μM of the selective oxytocin ligand [Thr4,Gly7] oxytocin (Perkin Elmer). Slides were quickly dipped in cold water, rapidly dried and developed for 90 hours using BioMax MR film (Kodak, Rochester, NY, USA). Autoradiogaphic ^125^I-receptor binding was quantified by using ImageJ measurement and specific binding was calculated by subtracting nonspecific binding from the total binding for each area. Three measures were averaged from different slices encompassing the specific brain region of interest and significance was determined by one-way ANOVA analysis. Adjacent slides were counterstained to aid in identification of brain structures.

### Behavioral tests

#### Animals

All animal procedures were in accordance with the National Institutes of Health Guide for the Care and Use of Laboratory Animals and were approved by the Icahn School of Medicine at Mount Sinai Institutional Animal Use and Care Committee. All mice used in behavioral experiments were exclusively from line 1 and sexually naïve, adult, male progeny of pairings of h,m/− and m/− mice on a C57BL/6J background. Mice were housed with two to four mixed genotype littermate animals per cage and maintained under a 12-hour light cycle (lights on, 07:00 to 20:00) in a controlled environment at a temperature of 23±1°C. Food and drinking water were provided *ad libitum*, except during the brief testing periods. Before each behavioral test, mice were acclimatized to the behavioral testing area for at least 60 minutes. All behavior testing occurred in the first half of the light part of the light–dark cycle. All behaviors were videotaped by a camera mounted above the apparatus and scored later by a single trained observer using a computer-assisted data acquisition system, Noldus Ethovision XT 8 (Leesburg, VA, USA).

#### Prepulse inhibition (PPI)

Sensorimotor gating was assessed using a PPI trial in which mice were tested in acoustically isolated startle chambers (MED Associates, St. Albans, VT, USA). The test started with a 10-minute acclimation followed by three sessions of trials. Background noise was 70 dB throughout the acclimation and three sessions of testing. In the first and last session, there were ten trials of startle stimuli alone (120 dB; 40 ms). The second, intermediate session consisted of 56 trials in which animals were presented with the startle stimulus alone or preceded by 100 ms with a 15-ms prepulse startle. The prepulse amplitude was 6, 8 or 12 dB above background with each repeated in random order. During this session, startle response magnitude, peak latency and onset latency to each stimulus were recorded. The startle response was defined as changes in force on the floor (i.e. ‘displacement’) between 30 and 70 ms after the onset of the startle stimulus. Animals that had a peak response in less than 20 ms or after 80 ms of the presentation of the stimuli were excluded. PPI was calculated as 100×(average startle amplitude across trials presenting prepulse and pulse/the average startle amplitude from trials in which the startle was presented alone).

#### Adult reciprocal social interaction

The reciprocal social interaction test was performed in a clear-walled, square open arena with dimensions measuring 28×28×20 cm (length, width, height). After two consecutive days of arena habituation, each subject was paired with an age- and weight-matched, unfamiliar C57BL6 male partner animal for 10 minutes. The time spent in social interaction (active contact such as sniffing, following and grooming the partner) was measured, and trials in which there were bouts of aggressive behavior were eliminated from analyses.

#### Novel object recognition and spatial relocation recognition

The object recognition tasks were conducted in a clear-walled, square arena with dimensions measuring 45×45×30 cm (length, width, height) and a visual cue (black and white striped pattern measuring 30×30 cm) was fixed on a wall directly outside of the apparatus in order to orient the animal to object locations and any spatial changes within the arena that were tested during the experiment. The experiment consisted of two habituation sessions, a familiarization session, a NOR test and an SSR test. After animals had undergone two 10-minute habituation sessions in the empty arena, they were allowed to freely explore two identical objects (object A), secured in fixed locations within the arena for a period of 10 minutes during the familiarization phase. During the NOR test, performed 1 hour later, one of the familiar objects was changed for another new object (object B), and the animals were allowed to explore for 5 minutes. During the SRR test, performed 10 minutes later, while object B was placed in the same location as in the previous NOR test, object A was moved to a novel location within the arena, and each subject was allowed to explore for 5 minutes. To control for odor cues, the arena and the objects were thoroughly cleaned between testing phases and subjects. Object investigation during NOR and SRR was defined as time spent touching, climbing over or sniffing the object (when the nose was oriented toward the object and the nose-object distance was 2 cm or less), whereas object investigation during the familiarization phase was used to identify innate side bias. The investigation ratio for NOR test was determined by B_time_/(A_time_+B_time_) and the SRR investigation ratio was determined by A_time_/(A_time_+B_time_) for each subject.

#### Three-chamber automated social behavior (social approach and preference for social novelty)

Social behavior was evaluated in a rectangular three-chambered box with each chamber measuring 20×40×22 cm (length, width, height). Dividing walls were made from clear Plexiglas, with small openings that allowed access into each chamber as mediated by sliding gates. During the habituation phase, the subject was allowed to explore the center chamber for 10 minutes and then all three chambers for another 10 minutes. Lack of innate side preference was confirmed during the second 10-minute habituation. For the social approach portion of the test, an unfamiliar male 129Sv/ImJ stimulus mouse (novel mouse) was introduced inside a weighted wire pencil cup in one side chamber with an empty pencil cup (novel object) in the opposite side chamber. The subject was allowed to explore all three chambers for 10 minutes. For the preference for social novelty portion of the test, 5 minutes later, the empty pencil cup was replaced by a second unfamiliar male 129Sv/ImJ stimulus mouse (novel mouse), with the original stimulus mouse now becoming a familiar social stimulus (familiar mouse). The subject was again allowed to explore all three chambers for 10 minutes. To control for odor cues, the arena and the objects were thoroughly cleaned between testing phases and subjects. A different stimulus mouse was used for each subject and although stimulus mice were re-used on different days, each was used only once per day. The time spent in each chamber and sniffing each pencil cup was later scored both manually and automatically by use of stringent software arena settings.

### Statistical analyses

Statistical analyses were conducted using SPSS 21.0 (SPSS Inc., Chicago, IL, USA) and graphed using GraphPad Prism 6.0 (La Jolla, CA, USA). mRNA levels and ligand-binding assay intensities were analyzed using Student’s independent *t*-test and one-way ANOVA. One-way ANOVA was used for analysis of the social interaction durations and investigation ratios during object recognition tasks. Within-group repeated measures ANOVA analyses were used to determine side preferences in the three-chamber automated social behavior testing. Two-way repeated measures ANOVA was used in analyses of prepulse inhibition. Post hoc comparisons were conducted with Fisher’s protected least significant difference tests following significant effects in the overall ANOVAs. The results are expressed as the means±s.e.m. A *P*-value of less than 0.05 was considered to be statistically significant. For all figures, *P<0.05, **P<0.01, ***P<0.001.

## Supplementary Material

Supplementary Material
