# Supplementary material for: Introduction of the human AVPR1A gene substantially alters brain receptor expression patterns and enhances aspects of social behavior in transgenic mice
Source: Dis Model Mech. 2014 Jun 12;7(8):1013–22. doi: 10.1242/dmm.017053 (PMC4107330; doi:10.1242/dmm.017053)
Supplement: Supplementary Material [file supp_7_8_1013__index.html]

Introduction of the human AVPR1A gene substantially alters brain receptor expression patterns and enhances aspects of social behavior in transgenic mice — Supplementary Material 

# Introduction of the human *AVPR1A* gene substantially alters brain receptor expression patterns and enhances aspects of social behavior in transgenic mice

## DMM017053 Supplementary Material

**Files in this Data Supplement:**

- **Supplementary Material**
