# Supplementary material for: Introduction of the human AVPR1A gene substantially alters brain receptor expression patterns and enhances aspects of social behavior in transgenic mice
Source: Dis Model Mech. 2014 Jun 12;7(8):1013–22. doi: 10.1242/dmm.017053 (PMC4107330; doi:10.1242/dmm.017053)
Supplement: Supplementary Material [file supp_7.8.1013_DMM017053.pdf]

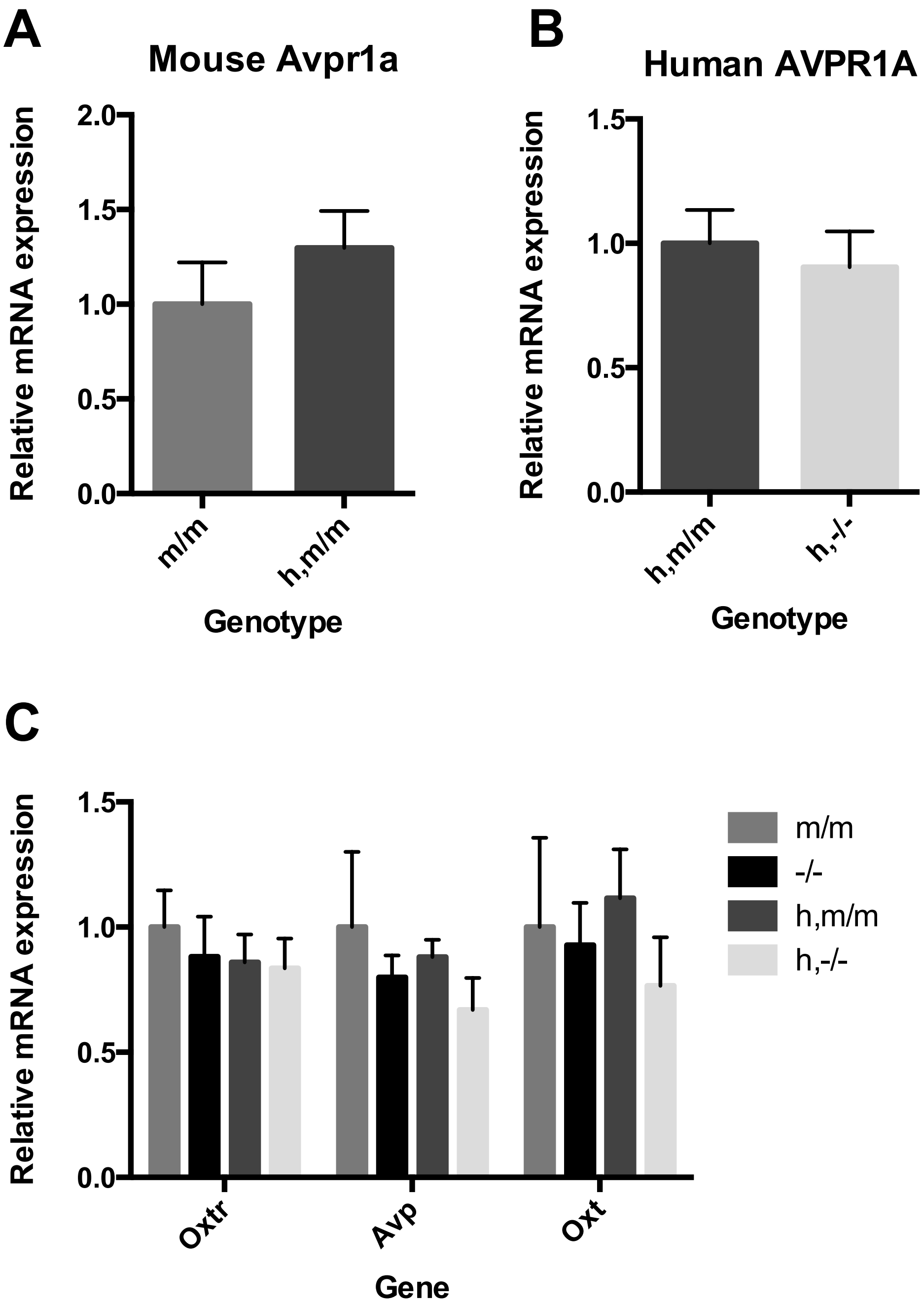

Supplementary Figure 1 - Verifying relative gene expression levels in transgenic animals. mRNA was isolated from left hemisphere brain samples of 6-month-old mice and used to synthesize cDNA which was subsequently analyzed via qRT-PCR. (A) Quantification of *mouse Avpr1a* mRNA expression in samples from m/m and h,m/m animals show that the levels of the endogenous gene is unaffected by the insertion of human *AVPR1A* gene (t-test, $p = 0.212$). (**B**) In humanized animals, the relative mRNA expression of human AVPR1A is similar across genotypes, regardless of the presence or absence of the murine *Avpr1a* gene (t-test, $p = 0.436$). (C) The mRNA expression levels of Avp, Oxt and Oxtr do not vary significantly between genotypes [n=6 per group; Oxtr: $F_{(3,24)} = 0.269, p = 0.847$; Avp: $F_{(3,24)} = 0.718, p = 0.551$; Oxt: $F_{(3,24)} = 0.402, p = 0.753$; One-way ANOVA]. Relative expression was obtained by normalizing to reference gene (*Gapdh)* from the same cDNA and results are expressed as a ratio of wildtype expression, resulting in an m/m ratio of 1. Data represent mean ± SEM.

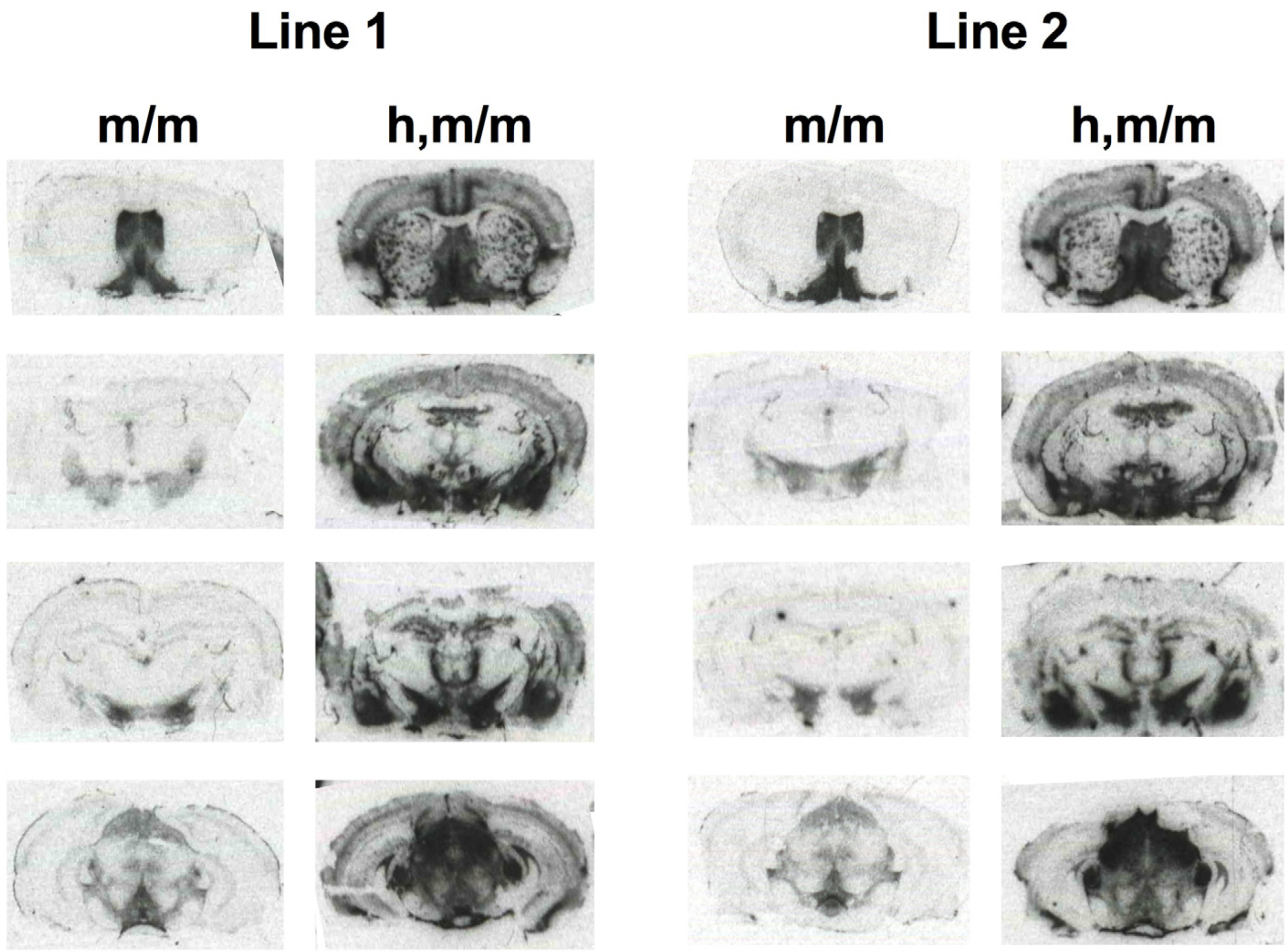

Fig. S2 – AVPR1A protein distribution pattern in the brains of transgenic animals from two independent lines. Radioactive AVP- I^{125} ligand binding was performed on fresh frozen coronally cut slide mounted sections of animals from the two independently derived BAC transgenic lines [m/m (n=2) and h,m/m (n=2) per line]. The developed film was pseudocolored, indicating binding intensity from low (white) to high (red). The binding intensities and distribution of the receptor was identical between the two lines. Line 1 is represented in the AVP- I^{125} ligand binding assay results of Figure 3 and this line was used in all biochemical and behavioural analyses.

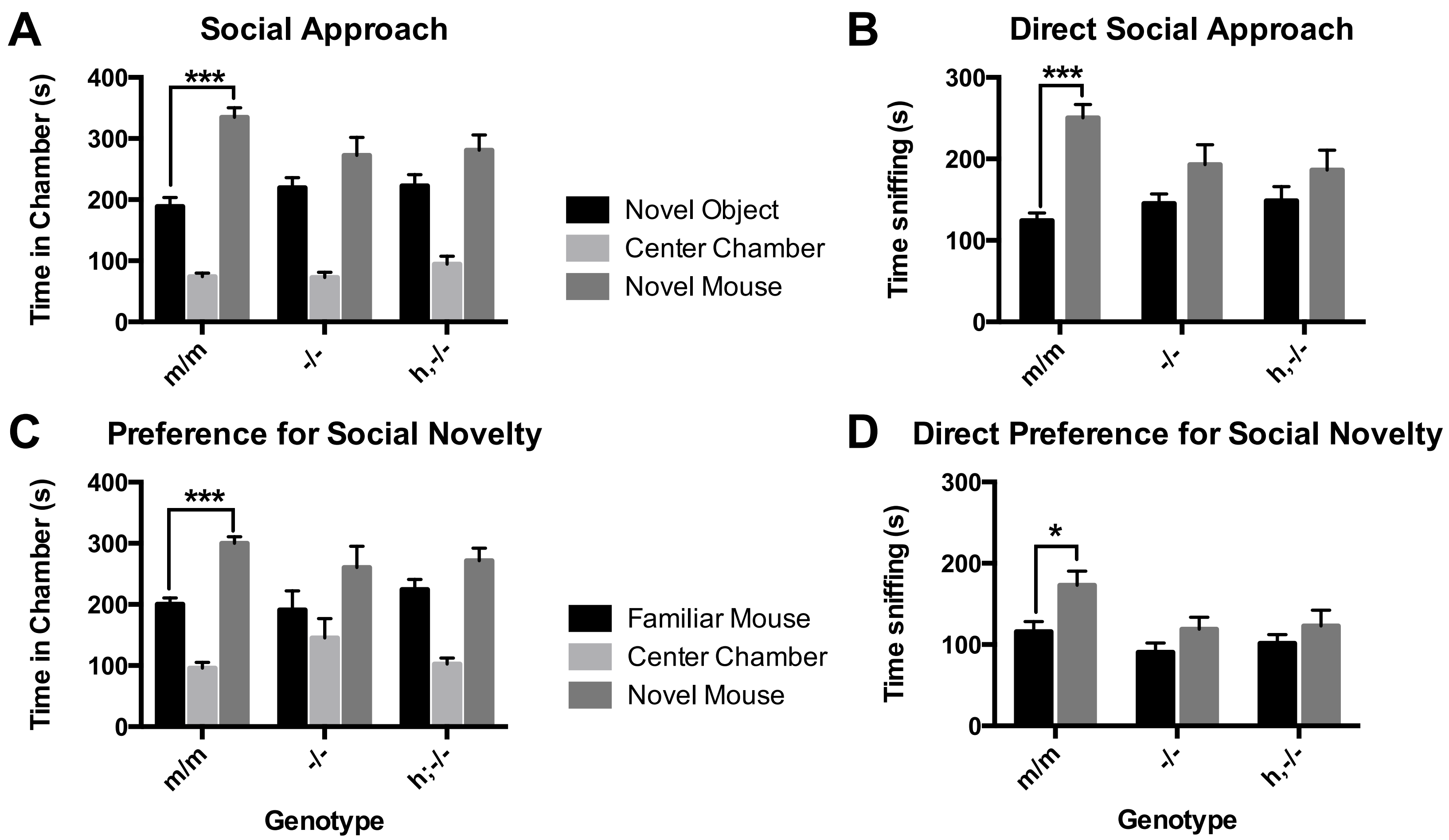

Fig. S3 – Automated 3-chamber apparatus analysis of Social Approach and Preference for Social Novelty.
(A) Mice [-/- (n=10), h,-/- (n=13), m/m (n=12)] were given the choice between a novel object and novel mouse to measure social approach. Wildtype mice spent significantly more time in the chamber containing the novel mouse as compared to the chamber containing the novel object while the knockout and humanized animal demonstrated no preference [m/m: $F_{(1,12)} = 23.627, p < 0.001$; -/-: $F_{(1,10)} = 1.455, p = 0.258$; h,-/- $F_{(1,13)} = 1.965$, p= 0.186]. (B) Closer measures of direct contact and sniffing of the novel mouse or object replicated these findings [m/m: $F_{(1,12)} = 29.366, p < 0.001$; -/-: $F_{(1,10)} = 4.816, p = 0.053$; h,-/- $F_{(1,13)} = 1.226$, p= 0.290] (C) Mice were then given a choice between a novel mouse and familiar mouse to measure preference for social novelty. Unlike wildtype animals, knockout mice do not show the normal significant preference for the chamber containing the novel mouse over familiar mouse [m/m: $F_{(1,12)} = 28.018, p < 0.001$; -/-: $F_{(1,10)} =$, $p =$; h,-/- $F_{(1,13)} = 2.522$, p= 0.138]. (D) Analysis of the time test animals spent sniffing each partner animal confirms this deficit in preference for social novelty in knockout animals that is not altered by introduction of the human transgene [m/m: $F_{(1,12)} = 8.818, p = 0.013$; -/-: $F_{(1,10)} = 3.811, p = 0.083$; h,-/- $F_{(1,13)} = 1.108$, p= 0.313]. . *p<0.05, ***p<0.001 from within-group repeated measures ANOVA. Data represent group mean $\pm$ SEM.

Brain Region	Primate AVPR1a	Rodent AVPR1a	h,-/-	m/m
Olfactory system				
Main olfactory bulb		+	+++	+
Accessory olfactory bulb		+	++	+
Anterior olfactory nucleus		+	-	-
Olfactory tubercle		+	+	+
Islands of Calleja	- to +	+ to ++	+	-
Cortical Areas				
Piriform cortex	- to +++	- to +	++	+
Entorhinal/perirhinal area	- to +++		+++	+
Insular cortex	- to ++		+++	-
Cingulate cortex	- to +++		+++	-
Parasubiculum and presubiculum	+++	+	+++	+
Dentate gyrus	+	+ to +++	+++	+
Basal Forebrain				
Lateral septual nucleus	++ to +++	+ to +++	-	+++
Medial septal nucleus	-	++	++	++
Bed nucleus of stria terminalis	+ to +++	+ to +++	+++	+
Basal Ganglia				
Caudate putamen	-		+	-
Ventral pallidum		++	-	++
Nucleus accumbens		++	++	++
Amygdala				
Central amygdaloid nucleus	+++	++	+++	+
Medial amygdaloid nucleus	++		+++	-

Lateral amygdaloid nucleus			+++	-
Basal amygdaloid nucleus	+		+++	-
Thalamus and hypothalamus				
Medial and lateral genticulate nucleus	-		+++	+
Lateral nuclei of thalamus	-		-	-
Ventral nuclei of thalamus	-		-	-
Anterior nuclei of thalamus	-	+	++	-
Midline nuclei of thalamus	+++	-	++	-
Center median parafascicular nuclei of thalamus	-		+++	-
Intralaminar nucleus of thalmus	+++		+++	-
Thalamic reticlar nucleus	-	+	-	-
Lateral preoptic area		+	++	+
Medial preoptic nucleus		-	++	-
Ventromedial hypothalamic nucleus	+		+	-
Suprachiasmatic nucleus		++	++	+
Paraventricular hypothalamic nucleus		++	+++	++
Supraoptic nucleus		-	++	-
Mammillary bodies	+++	++	+++	++
Tuberal nucleus		- to +++	+++	++
Zona incerta		+	+++	+
Brain Stem			+++	++
Substantia nigra pars compacta	+	- to +	++	+
Ventral tegmental area of colliculi		- to +	+++	+
Central grey		+ to ++	+++	+
Interpeduncular nucleus		++	+++	++
Median raphe nucleus			+++	++
Dorsal raphe nucleus		++	+++	++

Superior colliculus		++	+++	+
Parabrachial nucleus	++		++	-
Inferior olive nucleus	+	+++	++	+++
Pontine nuclei			+++	+
Locus coeruleus	+		++	-
Area postrema	+ to ++	+ to ++	++	++
Nucleus of solitary tract	+ to ++	+++	++	++
Spinal trigeminal nucleus	+ to ++		-	-
Pituitary gland				
Infundibulum	+++		+++	+

Table S1. Comparison of previous and current AVPR1A Radioligand Binding Assay Results.

This table shows regions of the brain and the relative levels of AVPR1A ligand binding densities. Primate binding data was gathered from rhesus monkey studies (Young 1999) and human studies (Loup 1991) while rodent data was gathered from rat (Tribollet 1988, Johnson 1993) and mouse studies (Dubois-Dauphin 1996) [47-50, 54]. A blank lane indicates that no information regarding binding was found in the literature, - means absent, + means weak, ++ means moderate and +++ means strong binding intensity (an indicator of receptor number and specific receptor binding affinity).
